# Evidence for scaling up HIV treatment in sub-Saharan Africa: A call for incorporating health system constraints

**DOI:** 10.1371/journal.pmed.1002240

**Published:** 2017-02-21

**Authors:** Evelinn Mikkelsen, Jan A. C. Hontelez, Maarten P. M. Jansen, Till Bärnighausen, Katharina Hauck, Kjell A. Johansson, Gesine Meyer-Rath, Mead Over, Sake J. de Vlas, Gert J. van der Wilt, Noor Tromp, Leon Bijlmakers, Rob M. P. M. Baltussen

**Affiliations:** 1 Department for Health Evidence, Radboud University Medical Center, Nijmegen, the Netherlands; 2 Department of Global Health and Population, Harvard T. H. Chan School of Public Health, Harvard University, Boston, Massachusetts, United States of America; 3 Department of Public Health, Erasmus MC, University Medical Center Rotterdam, Rotterdam, the Netherlands; 4 Africa Centre for Population Health, University of KwaZulu-Natal, Mtubatuba, South Africa; 5 Institute of Public Health, Heidelberg University, Heidelberg, Germany; 6 School of Public Health, Imperial College London, London, United Kingdom; 7 Department of Global Public Health and Primary Care, University of Bergen, Bergen, Norway; 8 Department of Addiction Medicine, Haukeland University Hospital, Bergen, Norway; 9 Center for Global Health and Development, Boston University, Boston, Massachusetts, United States of America; 10 Health Economics and Epidemiology Research Office, Department of Medicine, Faculty of Health Sciences, University of the Witwatersrand, Johannesburg, South Africa; 11 Center for Global Development, Washington, District of Columbia, United States of America; 12 Royal Tropical Institute (KIT), Amsterdam, the Netherlands

## Abstract

Jan Hontelez and colleagues argue that the cost-effectiveness studies of HIV treatment scale-up need to include health system constraints to be more informative.

**Summary points**

- The ever-growing HIV treatment programs in sub-Saharan Africa (SSA) present local policy makers with complex decision dilemmas, as international guidelines emphasize the need for expanded access to antiretroviral therapy (ART), yet funding has flatlined.
- We argue that the current evidence base for prioritizing ART scale-up strategies results in recommendations that are theoretically optimal but practically infeasible to implement.
- Cost-effectiveness analyses (CEAs) of scaling up ART in SSA should be made more responsive to the needs of policy makers by taking into account the local health system.
- We provide suggestions for a better integration of health system constraints into CEA by integrating supply- and demand-side constraints in mathematical models and improving the dialogue between researchers and policy makers.

## Introduction

The scale-up of antiretroviral therapy (ART) for HIV-infected people in sub-Saharan Africa (SSA) over the past 15 years is one of the most remarkable achievements in public health. With approximately 12 million people on treatment in 2015, life expectancy on the subcontinent has vastly improved [1]. Nevertheless, ART coverage in SSA is still suboptimal, HIV incidence remains high [1], and improved survival due to ART implies ever increasing numbers of people on treatment [2]. Substantial additional resources are needed to further scale up ART, yet funding has levelled off over the recent years, increasing the need for optimizing the allocation of limited resources [3].

The evidence base for resource allocation for strategies to scale up HIV treatment—e.g., on eligibility for treatment initiation—largely consists of cost-effectiveness analyses (CEAs) [4]. We reviewed the literature and found a total of 34 modelling studies performing prospective population-level CEAs on strategies of scaling up ART in SSA (S1 Text). Many of these strategies, e.g., universal testing and immediate treatment for all HIV-infected people, require substantial investments, yet most of the models implicitly assumed a well-functioning health system with unlimited resources by modelling immediate and sustainable implementation. In reality, most health systems experience constraints on the supply side (i.e., the capacity of the health system) and on the demand side (i.e., demand of HIV-infected people to receive care) (Box 1). In our review, no studies incorporated a comprehensive set of health system constraints. While 11 included some elements of demand-side constraints, only 4 incorporated some sort of supply-side constraint, and none incorporated a comprehensive set of both types of constraints.

**Box 1. Health system constraints**

***Supply-side constraints*** are defined as barriers in the capacity of the health system that hamper the delivery of care. For instance, drug stock outs at ART clinics in SSA are commonly reported, resulting in clinics being unable to distribute drugs to patients and thereby disrupting the continuum of care [5].

***Demand-side constraints*** are defined as barriers that affect the demand for care of people who would be eligible for a certain treatment or intervention. Commonly reported demand-side constraints in ART delivery are simply forgetting to visit the clinic or take medications, being away from home, changes in daily routine, stigma, or travel costs [6].

Here, we call for a more policy-relevant, integrated approach to CEA that incorporates health system constraints for two important reasons: (1) to determine more realistically the cost-effectiveness of the implementation of HIV treatment strategies within existing constraints and (2) to incorporate the resource needs, effects, and cost-effectiveness of overcoming constraints. For instance, health worker constraints could hamper the successful implementation of the WHO treatment recommendation of ART eligibility for all HIV-infected individuals, as the increase in eligible patients would necessitate more doctors and nurses. Implementation would require training of health workers, which introduces a time lag and requires financial investments, available human capital, and sufficient training capacity. CEA not performed in the context of these constraints may result in recommendations that are theoretically optimal but practically infeasible to implement [7]. In addition, the resources and time required to overcome constraints could be substantial and could make suggested HIV treatment strategies far less cost-effective than in hypothetical optimal settings of unlimited resources.

## Health system constraints

Supply-side constraints can be described in terms of the six health system building blocks as put forward by WHO [8]. These are (1) adequate *service delivery*—i.e., the availability of good, timely, and sustainable care to all patients along the full cascade of care; (2) a sufficient number of qualified *health workers* to provide treatment and care to all eligible patients; (3) a *health information system* that monitors health system performance and systematically records essential information on all patients—for example, to identify and respond to areas of underperformance or to improve re-enrolment of patients who defaulted treatment; (4) a reliable supply chain management of *medical products and technologies* to ensure sufficient supply of drugs and medical equipment; (5) sustainable *financing* to maintain care and treatment for those eligible; and (6) good *governance* that allows balanced, contextual decisions in allocating and managing limited resources.

Successful implementation of HIV treatment strategies is also determined by demand-side constraints. Stigma, limited knowledge about HIV, fatalism, out-of-pocket payments, and waiting times can affect health care-seeking behaviours such as consenting to testing, treatment uptake, and adherence. These constraints are all related to disease stage, as people who are at a relatively early stage of infection experience fewer symptoms and thus may be less motivated to seek care.

Supply- and demand-side constraints introduce complex, interrelated dynamics. Because of economies of scale and scope and interdependencies between supply and demand, changes in the number of people on ART rarely translate into a linear change in the need for financial, infrastructural, and human resources to provide the required services [9]. For instance, increased awareness of the benefits of HIV treatment—e.g., through visible improvements in the health of community members—may result in increased demand. In turn, increased demand may improve efficiency through economies of scale or result in overstretched resources and diseconomies of scale when resources are insufficient [9]. Furthermore, the relative impact of health system constraints is sensitive to time, context, and type of intervention. Constraints may change over time, e.g., the number of people on treatment may decrease as an effect of reduced incidence. Also, constraints are geographically heterogeneous, e.g., between rural and urban areas. Finally, certain health system constraints are more relevant to specific interventions; for example, community-based ART delivery requires different types of resources compared to clinic-based ART delivery. Because of these complex dynamics, constraints cannot be interpreted in isolation—for instance, as separate criteria in a policy process—but should be explicitly integrated within CEA.

## Moving forward

Some mathematical modelling studies have explored the effects of constraints on the cost-effectiveness of ART treatment strategies in SSA. Cleary et al., Fraser et al., and Anderson et al. determined the optimal treatment strategies within the available budgets for South Africa [10], Nigeria [11], and Kenya [12], respectively. Recently, Hontelez et al. determined the cost-effectiveness of ART for all HIV-infected people within different scenarios of supply- and demand-side constraints for ten countries in SSA [2]. Although we applaud these efforts, the extent to which they incorporated constraints is limited.

In order to move towards more realistic and policy-relevant CEAs, methodological innovations that include a more complete set of health system constraints into mathematical models are needed. Financial, infrastructural, and human resources required for alternative strategies need to be explicitly incorporated in a dynamic way. Supply-side constraints should not be treated statically—e.g., by simply assuming a maximum number of people who can receive ART. Rather, the effects of insufficient resources should dynamically translate into reduced performance along the full cascade of care in order to adequately reflect the patient- and population-level effects of policy decisions. For instance, the effect of undercapacity—i.e., when the number of people on treatment exceeds the capacity of the available health workers—could dynamically translate into lower adherence and higher attrition rates of people on ART. Likewise, demand-side dynamics along the full cascade of care should reflect the tendency of individuals to test, link, and adhere to treatment as a function of disease stage, health system access, and perceived quality of services.

We realize that the development of such models requires extensive efforts to improve empirical evidence of the underlying dynamics. Some descriptive data on health system constraints are available, and we know that poor adherence, unsuppressed viral loads, and high rates of attrition are signs of suboptimal treatment programs [13]. However, causal relationships describing the interactions between these constraints as well as their impact on the effectiveness of an HIV treatment program have yet to be quantified. For instance, the quantitative relationship between undercapacity and poor adherence is not yet fully understood. Reanalysis of existing data from longitudinal cohorts or including health system components in experimental designs could help to close this gap in knowledge. For instance, the ongoing cluster-randomized trials on treatment as prevention in SSA offer the opportunity to compare the outcomes of treatment programs among clinics with different capacities and among sites that use different treatment guidelines. Also, qualitative and quantitative data collection by interviewing patients could further increase the understanding of how supply-side dynamics and constraints affect patient behaviour.

Mathematical modelling also offers the opportunity to explore the impact of constraints even when precise estimates of the strengths of these relationships are unknown, e.g., through scenario and sensitivity analyses. These will be informative for policy makers by offering a range of expected outcomes under different constraints; in turn, they may incentivise further data collection by identifying key dynamic drivers that influence the outcome of CEAs. Future CEAs in HIV treatment and care should therefore include such explorative analyses, even when detailed data are not available.

Developing evidence that is more responsive to its decision context requires intensified collaboration between local policy makers and researchers. This is essential to formulate relevant research questions and assess what evidence is needed and which health system constraints would need to be taken into account. Finally, while improved CEA leads to a better evidence base for scaling up HIV treatment, it should be considered as only one input in the decision-making process. Other considerations, such as the preference of society to prioritise HIV treatment for the worse off or to reduce stigma, cannot be captured in a CEA. Fair decision-making processes, involving all relevant stakeholders, provide a means to take such considerations into account, in conjunction with evidence on cost-effectiveness [14].

## Conclusion

CEAs of scaling up HIV treatment in SSA should be made more responsive to the needs of policy makers by taking into account the local health system context. We call for a better integration of health system constraints into CEA—by accounting for such constraints in the use of mathematical models, facilitated by a dialogue between researchers and policy makers. This will result in CEA for HIV treatment strategies that are more policy relevant, allowing policy makers in SSA countries to make feasible choices for scaling up ART. Finally, lessons learned from improved CEAs in ART scale-up can also be applied to other disease areas that face similar dilemmas, such as malaria, tuberculosis, and noncommunicable diseases, and can help researchers and policy makers to better understand the impact of integration and decentralization of services across disease areas.

## Supporting information

S1 Text A systematic literature review of modelling studies performing prospective population level CEA of HIV treatment strategies in SSA. (DOCX)

## Abbreviations

ART: antiretroviral therapy
CEA: cost-effectiveness analysis
SSA: sub-Saharan Africa
