# Supplementary material for: Evidence for scaling up HIV treatment in sub-Saharan Africa: A call for incorporating health system constraints
Source: PLoS Med. 2017 Feb 21;14(2):e1002240. doi: 10.1371/journal.pmed.1002240 (PMC5319640; doi:10.1371/journal.pmed.1002240)
Supplement: S1 Text — (DOCX) [file pmed.1002240.s001.docx]

Supporting information for the manuscript: Evidence for scaling up HIV treatment strategies

in sub-Saharan Africa: A call for incorporating health system constraints

**A systematic literature review of modelling studies performing prospective population level cost-effectiveness analysis of HIV treatment strategies in sub-**

**Saharan Africa**

In support of our manuscript entitled “Evidence for prioritizing HIV treatment strategies in sub-Saharan Africa: A call for incorporating health system constraints”, we conducted a systematic review to assess the degree to which health system constraints have been included in the analysis of cost-effectiveness of antiretroviral treatment interventions.

We searched for articles published until November 2015 in five databases (Cochrane library, Web of Science, PubMed, Medline, Embase). Search terms included “cost-effective analysis”, “sub-Saharan Africa”, and “ART scale-up”, and yielded a total of 1800 original papers (S1 Figure). Two independent researchers screened the titles and abstracts of these papers for relevance. Articles were selected if written in English, and if a prospective CEA was performed on resource allocation at the general population level in sub-Saharan African countries. Retrospective cohort studies, patient models were excluded. The same researchers assessed in detail the full texts of 180 articles for final inclusion of 34 articles [[1-34](#_ENREF_1)] (S1 Figure).

S1 Figure: Selection process of included articles

¨

Search strategy

N = 1800

PreP

N = 22

PMTCT

N = 45

Selected articles based on

title/abstract

N = 180

Antiretroviral therapy

N = 113

Included

N = 34

Each article was assessed with respect to inclusion of health system constraints both on the supply-side (e.g., financial, human, or infrastructural resource constraints) and on the demand-side (e.g., health seeking behaviour, treatment adherence, or loss-to-follow-up rates). Inclusion of health system constraints were understood as incorporating the constraints in the analysis of the reported results. The results show that 11 articles incorporated one [[2](#_ENREF_2),[8](#_ENREF_8),[14](#_ENREF_14),[15](#_ENREF_15),[18](#_ENREF_18),[23](#_ENREF_23),[29](#_ENREF_29),[31](#_ENREF_31)] or several [[7](#_ENREF_7),[13](#_ENREF_13),[19](#_ENREF_19)] demand-side constraints) in the CEA. While most of these focused on lost to follow up [[2](#_ENREF_2),[7](#_ENREF_7),[8](#_ENREF_8),[13](#_ENREF_13),[15](#_ENREF_15),[18](#_ENREF_18),[19](#_ENREF_19),[23](#_ENREF_23),[29](#_ENREF_29)], others included adherence [[14](#_ENREF_14)], acceptance [[7](#_ENREF_7),[13](#_ENREF_13)] and health seeking behaviour [[19](#_ENREF_19),[31](#_ENREF_31)]. No article incorporated more than two demand-side constraints in the analysis. Supply-side constraints were only included in four of the articles of which all performed the CEAs within financial constraints [[10](#_ENREF_10),[19](#_ENREF_19),[24](#_ENREF_24),[30](#_ENREF_30)]. Only one article [[19](#_ENREF_19)] incorporated health system constraints on both the demand – i.e. in terms of lost to follow up and health seeking behaviour - and supply-side by assessing different financial constraints to the CEA.

S1 Table: Overview of articles including health system constraints in cost-effectiveness analysis of scaling up HIV treatment in sub-Saharan countries

| Year | Author | Title | Aim | Scenarios | Location | Health system constraints | |
| --- | --- | --- | --- | --- | --- | --- | --- |
|  |  |  |  |  |  | Demand-side constraints | Supply-side constraints |
| 2012 | Ventelou | The Macroeconomic Consequences of Renouncing to Universal Access to Antiretroviral Treatment for HIV in Africa: A Micro-Simulation Model | To assess the consequences of macroeconomic performance in respect to different HIV treatment strategies. | Freezing of ART programs to current level of access versus universal access (scaling up to 100% coverage by 2015 with two alternative ART strategies (CD4 ≤200 cells/µl and ≤350 cells/µl) | Cameroon, Tanzania and Swaziland |  |  |
| 2009 | Bikilla | Cost-effectiveness of anti-retroviral therapy at a district hospital in southern Ethiopia | To assess the cost-effectiveness of ART for routine clinical practice in a district hospital setting in Ethiopia. | ART at ≤200 cells/µl versus no ART | Ethiopia | X |  |
| 2011 | Chawana | Risk management in HIV/AIDS: ethical and economic issues associated with restricting HAART access only to adherent patients | To describe and quantify the economic consequences and discuss some ethical issues related to adherence and non-adherence to HAART from the provider’s perspective. | Adherent versus non-adherent patients | South Africa |  |  |
| 2006 | Cleary | The cost-effectiveness of Antiretroviral Treatment in Khayelitsha, South Africa – a primary data analysis | To estimate HIV healthcare utilisation, the unit costs of HIV services and the cost per life year (LY) and quality adjusted life year (QALY) gained of HIV treatment interventions from a provider's perspective. | ART at 200 cells/µl versus no ART | South Africa |  |  |
| 2006 | Marseille | The costs and benefits of private sector provision of treatment to HIV-infected employees in Kampala, Uganda | To determine the financial incentives of companies to treat HIV infected employees | No ART versus (a) CTX starting at WHO stage 2, (b) HAART+CTX starting at WHO stage 2, (c) "hybrid": CTX at WHO stage 2 and later HAART | Uganda |  |  |
| 2006 | Bachmann | Effectiveness and cost effectiveness of early and late prevention of HIV/AIDS progression with antiretrovirals or antibiotics in Southern African adults | To estimate the health effects, health service costs and incremental cost-effectiveness ratios of earlier or later use of antibiotics and ARV, alone and in combination in adult HIV infected people | (a) Antibiotics, (b) ART, (c) Antibiotics +ART | South Africa |  |  |
| 2012 | Palombi | Predicting Trends in HIV-1 Sexual Transmission in Sub-Saharan Africa Through the Drug Resource Enhancement Against AIDS and Malnutrition Model: Antiretrovirals for Reduction of Population Infectivity, Incidence and Prevalence at the District Level | Assess the reduction in incidence caused by ART | Universal coverage versus 45% treatment coverage in Malawi | Mozambique and Malawi | X |  |
| 2013 | Leisegang | A Novel Markov Model Projecting Costs and Outcomes of Providing Antiretroviral Therapy to Public Patients in Private Practices versus Public Clinics in South Africa | To compare the costs and outcomes of a private-care and a public-care ART program in South Africa. | Private-care versus public-care ART program | South Africa | X |  |
| 2012 | Mbonigaba | The Cost-effectiveness of Intervening in Low and High HIV Prevalence Areas in South Africa | To assess whether HIV/AIDS interventions could be more optimal in some areas of specific prevalence levels than in others | HIV/AIDS interventions in high versus low prevalence areas. | South Africa |  |  |
| 2015 | Fraser | Reorienting the HIV Response in Niger Toward Sex Work Interventions: From Better Evidence to Targeted and Expanded Practice | To assess the projected impact on the HIV epidemic of focused interventions on FSW | Focused FSW interventions versus other ART interventions | Nigeria |  | X |
| 2012 | Barnighausen | Economics of antiretroviral treatment vs. Circumcision for HIV prevention | To assess whether TasP is a game changer or if comparable benefits are obtainable at similar or lower costs by increasing coverage of medical circumcision (MMC) and ART at CD4 <350/μL | TasP versus a combination of male circumsision and ART at ≤350 cells/µl. | South Africa |  |  |
| 2011 | Hallett | Optimal Uses of Antiretrovirals for Prevention in HIV-1 Serodiscordant Heterosexual Couples in South Africa: A Modelling Study | To examine the impact and cost-effectiveness of earlier initiation to ART and/or PreP for HIV-1 prevention for disconcordant counples | ART at CD4 ≤350 cells/µl versus (a) ≤500 cells/µl, (b) ART in combination with PreP or (c) exclusive PreP to disconcordant counples. | South Africa |  |  |
| 2015 | Mitchell | Modelling the impact and cost-effectiveness of combination prevention amongst HIV serodiscordant couples in Nigeria | To estimate the impact and cost-effectiveness of treatment as prevention (TasP), pre-exposure prophylaxis (PrEP) and condom promotion for serodiscordant couples in Nigeria. | ART at ≤350 cells/µl versus (a) TasP, (b) short-term PrEP, (c) long-term PrEP, (d) condom promotion and (e) all combinations | Nigeria | X |  |
| 2012 | Mills | Earlier Initialization of Highly Active Antiretroviral Therapy Is Associated With Long-Term Survival and Is Cost-Effective: Findings From a Deterministic Model of a 10-Year Ugandan Cohort | To examine the cost-effectiveness of raising the eligibility treshold from 200 to 350 | ART at ≤200 cells/µl versus ≤350 cells/µl | Uganda | X |  |
| 2014 | Alistar | Comparative effectiveness and cost-effectiveness of antiretroviral therapy and pre-exposure prophylaxis for HIV prevention in South Africa | To study the population health outcomes and cost-effectiveness of implementing expanded ART coverage and oral PrEP in a setting with a heavy HIV burden. | ART at ≤350 cells/µl versus (a) universal access to ART, (b) PreP to general population and (c) PreP to high-risk population | South Africa | X |  |
| 2007 | Vijayaraghavan | Cost-Effectiveness of Alternative Strategies for Initiating and Monitoring Highly Active Antiretroviral Therapy in the Developing World | Determine the cost-effectiveness of initiating and monitoring highly active antiretroviral therapy (HAART) in developing countries according to developing world versus developed world guidelines. | Implementing developed versus developing world guidelines (ART at ≤350 cells/µl versus ≤200 cells/µl) | Developing countries |  |  |
| 2013 | Eaton | Health benefits, costs, and cost-eff ectiveness of earlier eligibility for adult antiretroviral therapy and expanded treatment coverage: a combined analysis of 12 mathematical models | Assess the potential health benefits, costs, and cost-effectiveness of various eligibility criteria for adult antiretroviral therapy andexpanded coverage | ART at ≤350 cells/µl versus (a) ART at ≤500 cells/µl and (b) ART for all HIV infected indiciduals | South Africa, Zambia, India, Vietnam | * | * |
| 2013 | Walensky | Cost-Effectiveness of HIV Treatment as Prevention in Serodescordant Couples | Compare the cost-effectiveness between early and late initiation to ART among serodisconcordant couples | Early versus late ART initiation | South Africa and India | X |  |
| 2011 | Hontelez | The Impact of the New WHO Antiretroviral Treatment Guidelines on HIV Epidemic Dynamics and Cost in South Africa | To estimate the long-term impact of the full WHO guidelines on the dynamics of the HIV epidemic and healthcare costs | ART at ≤200 cells/µl versus ≤350 cells/µl | The Hlabisa subdistrict of Umkhanyakunde in KZN, South Africa. | X | X |
| 2012 | Sempa | Cost-effectiveness of early initiation of first-line combination antiretroviral therapy in Uganda | To compare the cost-effectiveness of initiating cART in patients using the revised CD4 count threshold of 350 cells/μL as in the WHO 2010 guidelines versus (vs) cART initiation using a threshold of 250 cells/μL. | ART at ≤250 cells/µl versus ≤350 cells/µl | Uganda |  |  |
| 2006 | Badri | When to initiate highly active antiretroviral therapy in sub-Saharan Africa? A South African cost-effectiveness study | To assess the the impact of initiating therapy at CD4 >350/μl; 200–350/μl or <200/μl | No ART versus (a) ART at ≤350 cells/µl, (b) ART at 200–350 cells/µl and (c) ART at ≤200 cells/µl | South Africa |  |  |
| 2009 | Walensky | When to Start Antiretroviral Therapy in Resource-limited Settings | To assess the cost-effectiveness of when to start ART | No ART versus (a) ART at ≤250 cells/µl (or severe opportunistic disease) and (b) ART at ≤350 cells/µl (or severe opportunistic disease) | South Africa |  |  |
| 2011 | Granich | Expanding ART for Treatment and Prevention of HIV in South Africa: Estimated Cost and Cost-Effectiveness 2011-2050 | Assess the cost-effectiveness of expanded ART | ART at ≤200 cells/µl versus (a) ART at ≤350 cells/µl, (b) ART at ≤500 cells/µl and (c) ART at all CD4 levels | South Africa | X |  |
| 2014 | Anderson | Maximising the effect of combination HIV prevention through prioritisation of the people and places in greatest need: a modelling study | Assess the impact of focusing intervantions on geographies and key populations at high risk of HIV infection. | (a) Female sex workers versus (a) other women, (b) men who have sex with men and (c) other men | Kenya |  | X |
| 2006 | Goldie | Cost-Effectiveness of HIV Treatment in Resource-Poor Settings — The Case of Côte d’Ivoire | To assess the cost-effectiveness of no treatment, trimethoprim–sulfamethoxazole prophylaxis alone, antiretroviral therapy alone, and prophylaxis with antiretroviral therapy. | No treatment versus (a) trimethoprim–sulfamethoxazole prophylaxis alone, (b) antiretroviral therapy alone, and (c) prophylaxis with antiretroviral therapy. | Côte d’Ivoire |  |  |
| 2007 | Bishai | The cost effectiveness of antiretroviral treatment strategies in resource-limited settings | To compare costs and outcomes of different ART strategies with and without the availability of a second-line treatment regimen. | NO ART versus (a) syndromic management without laboratory tests (ART ONLY); (b) ART plus total lymphocyte counts every 6 months (TLC); (c) ART plus CD4 cell count assessment every 6 months (CD4); (d) ART plus CD4 cell count every 6months and viral load assessment 4weeks after the initiation of treatment, then every 6 months (VL) | Developing countries |  |  |
| 2005 | Hogan | Achieving the millennium development goals for health. Cost effectiveness analysis of strategies to combat HIV/AIDS in developing countries | To assess the costs and health effects of a range of interventions for preventing the spread of HIV and for treating HIV infected individuals | No intervention versus (a) mass media, (b) VCT, (c) Per education and treatment of STDs for CSW, (d) School based education, (e) treatment for STDs, (f) PMTCT, (g) HAART | East Africa and South East Asia |  |  |
| 2013 | Long | Portfolios of Biomedical HIV Interventions in South Africa: A Cost-Effectiveness Analysis | To evaluate the effectiveness and cost-effectiveness of combination biomedical HIV prevention and treatment scale-up | Expanded ART versus (a) screening and counselling, (b) voluntary male circumcision, (c) vaginal microbicide use, (d) oral PrEP | South Africa |  |  |
| 2015 | Ciaranello | Cost-effectiveness of first-line antiretroviral therapy for HIV-infected African children less than 3 years of age | To project the long-term clinical outcomes and cost-effectiveness of first-line nevirapine and lopinavir/ritonavir for HIV-infected children below 3 years of age. | No ART versus (a) first line nevirapine followed by second-line lopinovir/ritonavir and (b) first line lopinavir/followed by second line nevirapine | South Africa and Ivory Coast | X |  |
| 2008 | Cleary | Assessing efficiency and costs of scaling up HIV treatment | To develope an approach that simultaneously assess costs and efficiency | No ART versus (a) only first-line ART and (b) first-and second-line ART | South Africa |  | X |
| 2013 | Hontelez | Elimination of HIV in South Africa through Expanded Access to Antiretroviral Therapy: A Model Comparison Study | To understand the impact of UTT and the possibility of elimination of HIV based on implications of different model structures and assumptions. | ART at ≤350 cells/µl versus Universal test and treat (UTT) | South Africa | X |  |
| 2010 | Walensky | Scaling Up the 2010 World Health Organization HIV Treatment Guidelines in Resource-Limited Settings: A Model-Based Analysis | To determine which aspect of the new guidelines that should be implemented first in resource limited settings where immediate implementation of all the WHO trecommendations is unfeasible | No ART versus (a) ART at ≤200 cells/µl, (b) ART at ≤350 cells/µl, (c) multiple sequential ART regimens, (d) replacement of first/line stavudine with tenofovir. | South Africa |  |  |
| 2015 | Smith | Cost-Effectiveness of Antiretroviral Therapy and Isoniazid Prophylaxis to Reduce Tuberculosis and Death in People Living With HIV in Botswana | To examine the cost-effectiveness of IPT in Botswana, where antiretroviral therapy (ART) is widely available. | Comparison of seven strategies using a ombination of ART eligibility, provision of IPT and use of TST. | Botswana |  |  |
| 2010 | Dodd | Examining the Promise of HIV Elimination by ‘Test and Treat’ in Hyper-Endemic Settings | To investigate the impact of Test and Treat interventions under a range of epidemic contexts | Comparison of different test and treat interventions by altering the time since infection that treatment started. | Hyper endemic settings |  |  |

* The study includes several models, but do not specify whether health system constraints have been incorporated.

**REFERENCES**

1. Ventelou B, Arrighi Y, Greener R, Lamontagne E, Carrieri P, et al. (20121) The macroeconomic consequences of renouncing to universal access to antiretroviral treatment for HIV in Africa: a micro-simulation model. PLoS One 7: e34101.

2. Bikilla AD, Jerene D, Robberstad B, Lindtjorn B (2009) Cost-effectiveness of anti-retroviral therapy at a district hospital in southern Ethiopia. Cost Eff Resour Alloc 7: 13.

3. Chawana R, van Bogaert DK (2011) Risk management in HIV/AIDS: ethical and economic issues associated with restricting HAART access only to adherent patients. Afr J AIDS Res 10 Suppl 1: 369-380.

4. Cleary SM, McIntyre D, Boulle AM (2006) The cost-effectiveness of antiretroviral treatment in Khayelitsha, South Africa--a primary data analysis. Cost Eff Resour Alloc 4: 20.

5. Marseille E, Saba J, Muyingo S, Kahn JG (2006) The costs and benefits of private sector provision of treatment to HIV-infected employees in Kampala, Uganda. Aids 20: 907-914.

6. Bachmann MO (2006) Effectiveness and cost effectiveness of early and late prevention of HIV/AIDS progression with antiretrovirals or antibiotics in Southern African adults. AIDS Care 18: 109-120.

7. Palombi L, Bernava GM, Nucita A, Giglio P, Liotta G, et al. (2012) Predicting trends in HIV-1 sexual transmission in sub-Saharan Africa through the Drug Resource Enhancement Against AIDS and Malnutrition model: antiretrovirals for 5 reduction of population infectivity, incidence and prevalence at the district level. Clin Infect Dis 55: 268-275.

8. Leisegang R, Maartens G, Hislop M, Sargent J, Darkoh E, et al. (2013) A Novel Markov Model Projecting Costs and Outcomes of Providing Antiretroviral Therapy to Public Patients in Private Practices versus Public Clinics in South Africa. Plos One 8.

9. Mbonigaba J (2013) THE COST-EFFECTIVENESS OF INTERVENING IN LOW AND HIGH HIV PREVALENCE AREAS IN SOUTH AFRICA. South African Journal of Economic and Management Sciences 16: 183-198.

10. Fraser N, Kerr CC, Harouna Z, Alhousseini Z, Cheikh N, et al. (2015) Reorienting the HIV response in Niger toward sex work interventions: from better evidence to targeted and expanded practice. J Acquir Immune Defic Syndr 68 Suppl 2: S213-220.

11. Barnighausen T, Bloom DE, Humair S (2012) Economics of antiretroviral treatment vs. circumcision for HIV prevention. Proc Natl Acad Sci U S A 109: 21271-21276.

12. Hallett TB, Baeten JM, Heffron R, Barnabas R, de Bruyn G, et al. (2011) Optimal uses of antiretrovirals for prevention in HIV-1 serodiscordant heterosexual couples in South Africa: a modelling study. PLoS Med 8: e1001123.

13. Mitchell KM, Lepine A, Terris-Prestholt F, Torpey K, Khamofu H, et al. (2015) Modelling the impact and cost-effectiveness of combination prevention amongst HIV serodiscordant couples in Nigeria. Aids 29: 2035-2044.

14. Mills FP, Ford N, Nachega JB, Bansback N, Nosyk B, et al. (2012) Earlier initialization of highly active antiretroviral therapy is associated with long-term survival and is cost-effective: findings from a deterministic model of a 10-year Ugandan cohort. J Acquir Immune Defic Syndr 61: 364-369.

15. Alistar SS, Grant PM, Bendavid E (2014) Comparative effectiveness and cost-effectiveness of antiretroviral therapy and pre-exposure prophylaxis for HIV prevention in South Africa. BMC Med 12: 46.

16. Vijayaraghavan A, Efrusy MB, Mazonson PD, Ebrahim O, Sanne IM, et al. (2007) Cost-effectiveness of alternative strategies for initiating and monitoring highly active antiretroviral therapy in the developing world. J Acquir Immune Defic Syndr 46: 91-100.

17. Eaton JW, Menzies NA, Stover J, Cambiano V, Chindelevitch L, et al. (2013) Health benefits, costs, and cost-effectiveness of earlier eligibility for adult antiretroviral therapy and expanded treatment coverage: a combined analysis of 12 mathematical models. Lancet Glob Health 2: 23-34.

18. Walensky RP, Ross EL, Kumarasamy N, Wood R, Noubary F, et al. (2013) Cost-effectiveness of HIV treatment as prevention in serodiscordant couples. N Engl J Med 369: 1715-1725.

19. Hontelez JA, de Vlas SJ, Tanser F, Bakker R, Barnighausen T, et al. (2011) The impact of the new WHO antiretroviral treatment guidelines on HIV epidemic dynamics and cost in South Africa. PLoS One 6: e21919.

20. Sempa J, Ssennono M, Kuznik A, Lamorde M, Sowinski S, et al. (2012) Cost-effectiveness of early initiation of first-line combination antiretroviral therapy in Uganda. BMC Public Health 12: 736.

21. Badri M, Cleary S, Maartens G, Pitt J, Bekker LG, et al. (2006) When to initiate highly active antiretroviral therapy in sub-Saharan Africa? A South African cost-effectiveness study. Antivir Ther 11: 63-72.

22. Walensky RP, Wolf LL, Wood R, Fofana MO, Freedberg KA, et al. (2009) When to start antiretroviral therapy in resource-limited settings. Ann Intern Med 151: 157-166.

23. Granich R, Kahn JG, Bennett R, Holmes CB, Garg N, et al. (2012) Expanding ART for treatment and prevention of HIV in South Africa: estimated cost and cost-effectiveness 2011-2050. PLoS One 7: e30216.

24. Anderson SJ, Cherutich P, Kilonzo N, Cremin I, Fecht D, et al. (2014) Maximising the effect of combination HIV prevention through prioritisation of the people and places in greatest need: a modelling study. Lancet 384: 249-256.

25. Goldie SJ, Yazdanpanah Y, Losina E, Weinstein MC, Anglaret X, et al. (2006) Cost-effectiveness of HIV treatment in resource-poor settings - The case of Cote d'Ivoire. New England Journal of Medicine 355: 1141-1153.

26. Bishai D, Colchero A, Durack DT (2007) The cost effectiveness of antiretroviral treatment strategies in resource-limited settings. Aids 21: 1333-1340.

27. Hogan DR, Baltussen R, Hayashi C, Lauer JA, Salomon JA (2005) Achieving the millennium development goals for health - Cost effectiveness analysis of strategies to combat HIV/AIDS in developing countries. British Medical Journal 331: 1431-1435.

28. Long EF, Stavert RR (2013) Portfolios of biomedical HIV interventions in South Africa: a cost-effectiveness analysis. J Gen Intern Med 28: 1294-1301.

29. Ciaranello AL, Doherty K, Penazzato M, Lindsey JC, Harrison L, et al. (2015) Cost-effectiveness of first-line antiretroviral therapy for HIV-infected African children less than 3 years of age. Aids 29: 1247-1259.

30. Cleary SM, McIntyre D, Boulle AM (2008) Assessing efficiency and costs of scaling up HIV treatment. Aids 22 Suppl 1: S35-42.

31. Hontelez JA, Lurie MN, Barnighausen T, Bakker R, Baltussen R, et al. (2013) Elimination of HIV in South Africa through expanded access to antiretroviral therapy: a model comparison study. PLoS Med 10: e1001534.

32. Walensky RP, Wood R, Ciaranello AL, Paltiel AD, Lorenzana SB, et al. (2010) Scaling Up the 2010 World Health Organization HIV Treatment Guidelines in Resource-Limited Settings: A Model-Based Analysis. Plos Medicine 7.

33. Smith T, Samandari T, Abimbola T, Marston B, Sangrujee N (2015) Implementation and Operational Research: Cost-Effectiveness of Antiretroviral Therapy and Isoniazid Prophylaxis to Reduce Tuberculosis and Death in People Living With HIV in Botswana. J Acquir Immune Defic Syndr 70: e84-93.

34. Dodd PJ, Garnett GP, Hallett TB (2010) Examining the promise of HIV elimination by 'test and treat' in hyperendemic settings. Aids 24: 729-U128.
